# A Novel tRF-Lys-TTT-012 in Qingyu Pigs Mediates the Conversion of Muscle Fibers from Fast-Twitch to Slow-Twitch Type

**DOI:** 10.3390/ani15203044

**Published:** 2025-10-20

**Authors:** Kai Wang, Jiaxin Li, Yuhang Lei, Xinyi Wang, Dujun Chen, Mailin Gan, Li Zhu, Linyuan Shen

**Affiliations:** 1 Farm Animal Germplasm Resources and Biotech Breeding Key Laboratory of Sichuan Province, Sichuan Agricultural University, Chengdu 611130, China; 2023202026@stu.sicau.edu.cn (K.W.); lijiaxin1@stu.sicau.edu.cn (J.L.); leiyuhang0819@stu.sicau.edu.cn (Y.L.); 2024202054@stu.sicau.edu.cn (X.W.); 2024202034@stu.sicau.edu.cn (D.C.); ganmailin@sicau.edu.cn (M.G.); 2 State Key Laboratory of Swine and Poultry Breeding Industry, Sichuan Agricultural University, Chengdu 611130, China; 3 Key Laboratory of Livestock and Poultry Multi-Omics, Ministry of Agriculture and Rural Affairs, College of Animal and Technology, Sichuan Agricultural University, Chengdu 611130, China

**Keywords:** skeletal muscle fiber type, tsRNA, mitochondria

## Abstract

**Simple Summary:**

Skeletal muscle is composed of various types of muscle fibers, and the composition of these fibers plays a significant role in muscle health, meat quality, and flavor. However, the mechanisms underlying tsRNA regulation of muscle fiber type conversion remain unclear. In this study, we used the psoas major muscle and latissimus dorsi muscle of pigs as models to reveal the expression profiles of tsRNAs in muscles with different fiber compositions. The results indicate that tsRNAs play a crucial regulatory role in muscle fiber type conversion. This study provides new insights into the molecular regulatory mechanisms of muscle fiber type conversion and offers references for the treatment and improvement of meat quality.

**Abstract:**

Skeletal muscle, the largest organ within the animal body, consists of multiple muscle fiber types. The distribution of these fiber types significantly impacts both athletic performance and the quality of meat. Growing evidence has demonstrated that transfer RNA (tRNA)-derived small RNAs (tsRNAs) are not merely byproducts of tRNA metabolism but also participate in multiple cellular metabolic processes. However, the role of tsRNAs in skeletal muscle fiber type transition remains elusive. In this study, a total of 403 differentially expressed tsRNAs were identified through small RNA sequencing in psoas major muscle (PM) and latissimus dorsi muscle (LD), among which 220 tsRNAs including tRF-Lys-TTT-012 were upregulated in psoas major muscle. Functional studies in C2C12 and PK15 cells demonstrated that it inhibited the proliferative capacity of C2C12 cells while promoting myogenic differentiation, increased the proportion of slow muscle fibers after differentiation, and drove muscle fiber type transition toward slow fibers. Additionally, tRF-Lys-TTT-012 enhanced mitochondrial number and function, potentially linking to the promotion of slow fiber characteristics. Collectively, tRF-Lys-TTT-012 may serve as a promising marker for slow muscle fibers and uncover a novel potential target for skeletal muscle fiber type transition toward the slow fiber phenotype.

## 1. Introduction

Skeletal muscle has significant importance in both human and domestic animals, playing a crucial role in their overall physiology, including locomotion, postural maintenance, metabolism and nutrient storage. Additionally, it contributes substantially to the economic worth of livestock, constituting around 40% of the live weight of these livestock. Skeletal muscle consists primarily of elongated, parallel, cylindrical muscle fibers that are interconnected by a sophisticated and organized network of intramuscular membrane and basement membrane [1]. Skeletal muscle fibers have been classified according to the different MYHC isoforms expressed by different muscle fibers, and the most commonly used classifications include four fiber types: type I (slow oxidative: slow single contraction, oxidative metabolism feeds, fatigue-resistant), IIa (fast oxidative: faster single contraction, oxidative metabolism feeds, intermediary metabolic properties), IIb (fast enzyme-mediated: fastest single contraction, glycolytic metabolism, susceptible to fatigue) and IIX intermediate [2,3]. The disparity in performance between type I and type II muscle fibers can be attributed to the disparity in mitochondrial content. Type I muscle fibers possess a higher concentration of mitochondria, enabling them to produce more adenosine triphosphate (ATP) through oxidative phosphorylation. Consequently, type I fibers exhibit greater resistance to fatigue compared to type II fibers, despite their slower contraction rate [4]. Skeletal muscle fiber type conversion requires intricate and highly coordinated biochemical mechanisms and pathways, such as signaling transduction, nutritional regulation, transcription factors control, hormone regulation and mitochondrial biogenesis [5,6,7,8].

tsRNAs are non-coding small RNAs that are specifically cleaved segments derived from mature tRNAs or precursor tRNAs [9,10,11]. Based on the biological origin of tRNAs and the naming of their breakpoints, they are usually categorized into two types: tRNA-halves and tRNA-derived fragments (tRFs) [12,13]. There is mounting evidence indicating that tsRNAs exert their biological functions through a diverse array of mechanisms, including protein or mRNA interactions [14], gene expression regulation [15,16,17], cell cycle control [18], and epigenetic modifications [19]. With the deepening of the research of tsRNAs, more and more evidence that tsRNAs has played a key role in the economic characters of livestock. Recently Gan et al. sequenced tsRNAs from skeletal muscle of normal and intrauterine growth retarded pigs and found that 38 specifically expressed in IUGR pigs, and the target genes of these differentially expressed tsRNAs were highly enriched in metabolic pathways [20]. Multiple differential tsRNAs were also found in the adipose tissue of obese and lean pigs, which are indirectly involved in the regulation of bioenergetic metabolism [21]. However, few findings have shown that tsRNAs may be key targets in the regulation of skeletal muscle development or muscle fiber transformation.

Based on previous studies showing the involvement of non-coding RNAs in muscle fiber type differentiation, we hypothesize that the tRF-Lys-TTT-012 plays a crucial role in regulating muscle fiber type transition, particularly in slow-twitch fibers, through modulation of the cell cycle and mitochondrial function.

In this study, our objective was to elucidate the role and underlying mechanism of tsRNAs in the process of skeletal muscle fiber type conversion. We initially observed an enrichment of tRF-5C in the latissimus dorsi (LD) and psoas major (PM) muscles. Based on the sequencing results, tRF-Lys-TTT-012 was subsequently selected for functional validation, considering factors such as Log2FC, *p*-value, Q-value, and CPM value of this specific type of tsRNAs in both muscles. We determined that overexpression of tRF-Lys-TTT-012 leads to decreased proliferation and increased differentiation of C2C12 myoblasts, inducing mitochondrial ontogeny and myofiber type conversion. Consequently, the expression of genes involved in the development and proliferation of C2C12 myoblasts is changed. These results imply that tRF-Lys-TTT-012 precisely controls the proliferation and differentiation of C2C12 myoblasts.

## 2. Materials and Methods

### 2.1. Experimental Animals

A total of 9 commercial pigs were included in this study: 3 healthy Qingyu pigs weighing 95.33 ± 2.08 kg were used for small RNA sequencing and sequencing validation, 3 Duroc × Landrace × Yorkshire (DLY) and 3 Chenghua (CH) pigs were used for qRT-PCR. All animals were fasted for 24 h before slaughter but had free access to drinking water. Pigs were euthanized by electrical stunning. Post blood removal, samples (2 g) were taken from the middle part of PM and LD at the last rib, frozen in liquid nitrogen, and stored at 80 °C for further use. All animal experimental procedures were formally approved by the Animal Ethics and Welfare Committee of Sichuan Agricultural University, Chengdu, China (Approval number 2021302137, approved in 14 March 2021).

### 2.2. tRF & tiRNA Sequencing

Total RNA was extracted from muscle samples using RNAiso Plus (9109, Takara, Kusatsu, Japan), and pretreated with RNA Pretreatment (Arraystar, Rockville, MD, USA) to remove modifications, including 3′-aminoacyl, 2′,3′-cyclic phosphate, 5′-OH, m1A, and m3C. For standard small RNA sequencing on Illumina NextSeq instrument, the sequencing type is 50 bp singleread. Total RNA of each sample was sequentially ligated to 3′ and 5′ small RNA adapters. cDNA was then synthesized and amplified using Illumina’s proprietary RT primers and amplification primers. Subsequently, approximately 134–160 bp PCR amplified fragments were extracted and purified from the PAGE gel. And finally, the completed libraries were quantified by Agilent 2100 Bioanalyzer (Agilent, Santa Clara, CA, USA). Diluted libraries were loaded onto the kit and forwarded for sequencing on an Illumina NextSeq 500 system (Illumina, San Diego, CA, USA). The tsRNA sequencing data were stored at the National Genomics Data Center (NGDC).

### 2.3. Cell Culture

C2C12 myoblasts and PK15 porcine kidney cells were purchased from NICR (Beijing, China). Proliferative cell culture was performed in DMEM medium containing 10% fetal bovine serum (Gibco, Waltham, MA, USA) and 1% penicillin-streptomycin (Gibco, USA). At 80% confluence, cells were cultured for 6 days using a differentiation medium, which consisted of DMEM supplemented with 2% horse serum, to induce the formation of myotubes. PK15 porcine kidney cells were cultured in DMEM (Hyclone, Seattle, WA, USA) supplemented with 10% fetal bovine serum (Gibco, USA) and 1% penicillin–streptomycin (Gibco, USA). All cells were cultured at 37 °C with 5% CO_2_.

### 2.4. Cell Transfection of tRF-Lys-TTT-012 Mimics

The exogenous tRF-Lys-TTT-012 mimics were synthesized at Genepharma (Shanghai, China). C2C12 myoblasts and PK15 porcine kidney cells were inoculated in 12-well plates at a density of 2 × 10^5^ cells/mL and cultured for 24 h. The exogenous tRF-Lys-TTT-012 mimics were transfected into C2C12 and PK15 cells at a final concentration of 20 nM. Transfection was performed using the Lipofectamine 3000 (Invitrogen, Carlsbad, CA, USA) liposome transfection method as described in the manufacturer’s instructions. Sequences of exogenous tRF-Lys-TTT-012 mimics were shown in Table 1.

### 2.5. Quantitative Real-Time PCR (qRT-PCR)

According to manufacturer’s instructions, total RNA of muscle tissue samples and cell samples were extracted with RNAiso Plus (Takara, Japan). Then, RNA concentrations in different samples were detected in NanoDrop 2000 (Thermo Fisher Science, Waltham, MA, USA). Reverse transcription of mRNA was performed using the PrimeScriptTM RT reagent kit (TaKaRa, Japan), and the Mir-XTM miRNA First-Strand Synthesis Kit (TaKaRa, Japan) for tsRNA. The mRNA cDNA synthesis program was 42 °C for 2 min, 37 °C for 15 min, and 85 °C for 5 s. The tsRNA cDNA synthesis program was 1 h at 37 °C and 5 min at 85 °C. The cDNA was subjected to real-time quantitative polymerase chain reaction with TB Green PreMix EX TaqTM II (Takara, Japan) in the Bio-Rad CFX96 Real-Time PCR Detection System (Bio-Rad, Hercules, CA, USA). The internal reference genes for mRNA are GAPDH and β-actin, and the internal reference gene for tsRNA is U6. The relative expression levels of mRNA and tsRNA were calculated using the 2^−∆∆Ct^ method. All gene expressions were normalized. The primer sequences used during the qRT-PCR experiments were shown in Table 2.

### 2.6. Cell Proliferation Assay

The cell proliferative activity was assessed after transfecting C2C12 myoblasts with exogenous mimics for 24 h. the cell counting kit-8 (CCK-8) was purchased from Beyotime (Shanghai, China) and the 5-ethynyl-2′-deoxyuridine (Edu) kit purchased from RIBOBIO (Guangzhou, China). C2C12 myoblasts were treated with 10 μL CCK-8 solution in 96-well plates and incubated for 1.5 h before measuring the absorbance at 450 nm using the Varioskan LUX Multifunctional Enzyme Labeler (Thermo Fisher, USA). The CCK-8 assay was used to detect the proliferative activity of cells at 0 h, 24 h, 48 h and 72 h time points. The EDU assay was performed in accordance with the manufacturer’s instructions and positive cells were visualized under a fluorescence microscope after staining.

### 2.7. Western Blot Analysis

Cells were lysed with RIPA lysis solution (Beyotime, China) containing 50 mM Tris (pH 7.4), 150 mM NaCl, 1% Triton X-100, 1% sodium deoxycholate, 0.1% SDS, and 1 mM PMSF (Beyotime, China) for 30 min. Then, the lysed samples were centrifuged in a centrifuge at 4 °C, 15,000 rpm for 15 min. Protein concentration in individual samples was measured using the BCA protein concentration kit (Beyotime, China). Proteins were separated on sodium dodecyl sulfate-polyacrylamide gels (SDS-PAGE) and transferred to PVDF membranes. The primary antibodies were incubated overnight at 4 °C and secondary antibodies were incubated for 1 h at room temperature. Target protein were detected with PGC1 alpha antibody at 1:1000 concentration (Novus Biologicals, Centennial, CO, USA). Protein band images were processed and analyzed using ImageJ software (1.52a version, NIH, Bethesda, MD, USA).

### 2.8. Immunofluorescence Staining

C2C12 myoblasts in 12-well plates were fixed with 4% polymethanol for 1 h at room temperature after the treatment was completed. After fixation, the cells were permeabilized with 0.02% Triton X-100 for 30 min to solubilize lipids and enhance the antibody’s permeability into the cell membrane. To minimize nonspecific binding, cells were incubated with antibodies after blocking with 10% goat serum for 30 min. After blocking was completed, the cells were incubated overnight at 4 °C with primary antibody (1:100) and 1 h at room temperature with secondary antibody (1:500). Cell nuclei were stained with DAPI (Beyotime, China) for 20 min at room temperature.

### 2.9. Measuring Mitochondrial Activity

The Mito-Tracker Red CMXRos kit (Beyotime, China) was used to detect mitochondrial membrane potential in this experiment. The mitochondrial stain was diluted with DMEM serum-free medium at a ratio of 1:1000 according to the manufacturer’s instructions. The diluted mitochondrial staining solution was added to cells cultured in 12-well plates and incubated for 30 min. After incubation, the cells were washed, and the stained mitochondria were observed under a fluorescence microscope. Fluorescence images were analyzed using ImageJ software (1.52a version, USA).

### 2.10. Reactive Oxygen Species (ROS) Staining Assay

In this experiment, the Reactive Oxygen Species (ROS) Detection Reagents kit (Thermo Fisher, USA) was used to detect ROS in cells, which provided derivatives of reduced fluorescein and calcineurin as indicators of cell permeation containing reactive oxygen species. The cells were separated from the growth medium by centrifugation and then resuspended in pre-warmed PBS buffer solution containing the probe, followed by a 30 min incubation period. After incubation, the loading buffer was removed, and the cells were washed with PBS. Subsequently, the cells were observed under a fluorescence microscope. Fluorescence images were analyzed using ImageJ software (1.52a version, USA).

### 2.11. Mitochondrial DNA Copy Number Assay

Mitochondrial DNA copy number was quantified by the real-time-PCR based method using a mitochondrial DNA copy number assay kit (MCN2; Detroit R&D, Detroit, MI, USA) as per the manufacturer’s instructions. Reactions were performed with 10 ng of DNA, and mitochondrial DNA copy numbers were normalized with nuclear DNA copy number using the 2^−ΔΔCT^ method.

### 2.12. Flow Cytometry

C2C12 myoblasts were harvested by trypsinization and centrifuged at 1000 r/min for 5 min (approximately 6 × 10^5^ cells were collected) after transfecting for 24 h. The cells were then resuspended in 2 mL of 4 °C 70% ethanol and incubated for an additional 30 min on ice. Next, 2 mL of 4 °C PBS solution was added, and the mixture was vortexed and centrifuged at 300 g/min for 5 min, with this step repeated. Afterward, 500 μL of PI/Rnase staining buffer (BD, Franklin Lakes, NJ, USA) was added, and the cells were incubated at 4 °C for 30 min. Following the incubation, 2 mL of PBS solution was added to wash the cells once, and then cells were resuspended in 400 μL of PBS solution. Finally, cell detection was performed online using the CytoFLEX flow cytometer (Backman, Atlanta, GA, USA), and the data were analyzed using Modfit LT software (6.0.11, USA).

### 2.13. Bioinformatics Analysis

Visualization of raw tRF & tiRNA sequencing data generated by the NextSeq 500 Sequencing System using the Bioinformatics Visualization website (https://www.bioinformatics.com.cn/, accessed on 14 October 2025). In the R environment, online resources such as TargetScan (https://www.targetscan.org/, accessed on 14 October 2025) and miRDB (http://www.mirdb.org/, accessed on 14 October 2025) were used for the purpose of predicting target genes of tRF-Lys-TTT-012. Functional enrichment of target genes by MetaScape and analysis of signaling pathways involved in target genes using KEGG pathway. RNAfold WebServer (http://rna.tbi.univie.ac.at/cgi-bin/RNAWebSuite/RNAfold.cgi, accessed on 14 October 2025) was used to predict the shear site of tRF-Lys-TTT-012.

### 2.14. Statistical Analysis

If each experiment was not explicitly labeled in the text, three biological replicates were performed for each experiment (*n* = 3). The results were expressed as the mean ± standard error of the mean (SEM). Data analysis was performed using GraphPad Prism 8.0.2 software (San Diego, CA, USA). Differences between two groups were compared using the paired two-tailed Student’s *t*-test. *p* < 0.05 was regarded as a significant value.

## 3. Results

### 3.1. Differences in Muscle Fiber Type Composition Between LD and PM in Pigs

**Figure 1:**
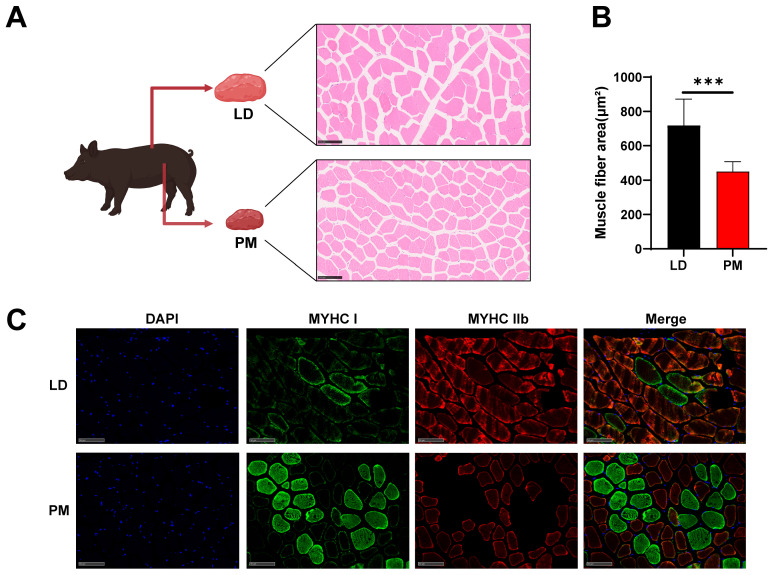
Analysis of the tsRNA composition in LD and PMs. (**A**) There are significant differences between the LD and PM tissues in pigs, and tsRNA sequencing was performed to analyze them. HE staining of cross-sections from LD and PM tissues. (**B**) Quantitative comparison of the cross-sectional area of muscle fibers in HE-stained sections. (**C**) Immunofluorescence staining of MYHC I and MYHC IIb in LD and PM tissues. Scale bar represents 100 μm. All data were presented as mean ± SEM. The symbol * denotes a significance level of * *p* < 0.05, ** *p* < 0.01, *** *p* < 0.001.

There were significant differences in appearance, meat color, and pH between the LD and PMs, with the cross-sectional area of muscle fibers in LD being significantly larger than that in PM (Figure 1A,B). These differences are associated with the composition of muscle fiber types (Figure 1C). Here, we aim to further elucidate the underlying causes of these differences through tsRNA-Seq (Figure 1A).

### 3.2. Types of tRFs and tiRNAs Are Differentially Expressed in LD and PM

**Figure 2:**
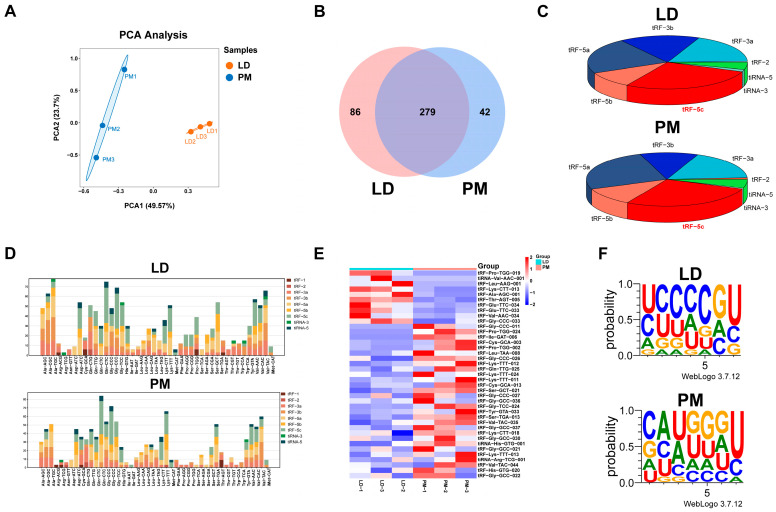
The overview of tRFs and tiRNA expression in the LD and PMs. (**A**) The principal component analysis (PCA) of tRFs and tiRNAs expressions in LD and PM samples. (**B**) Venn diagram of tRFs and tiRNAs expression in muscle samples from both groups. (**C**) Distribution of different types of tRF and tiRNA in two groups of muscle samples. (**D**) Stacked plot of all isoforms of tRFs and tiRNAs clustered by tRNA anticodons. The *x*-axis represents tRNAs with the same anticodon, while the *y*-axis shows the number of all isoforms of tRFs and tiRNAs originating from the respective anticodon tRNA. (**E**) The clustering heatmap of differentially expressed tsRNAs in samples of the LD and PM. (**F**) Seed sequence characteristics of differentially expressed tsRNAs.

To further investigate the potential key regulatory role of tsRNAs in muscle development and myofiber type transformation, we collected the LD and PMs from three pigs for tRFs & tiRNAs sequencing experiment. A total of 649 tsRNAs were identified in both muscle tissues, and the PCA revealed an ideal correlation between the tsRNAs of the two muscle tissue samples (Figure 2A). Among them, 279 co-expressed tRFs and tiRNAs were detected in the LD and PM groups, with 86 being specifically expressed in the LD group and 42 in the PM group (Figure 2B). The subtype distribution of differentially expressed tsRNAs in the LD and PMs was analyzed using a pie chart. Additionally, the number of tsRNA subtypes derived from tRNAs with the same anticodon was quantified using a stacked plot. Notably, the tRF-5c subtype exhibited the largest proportion in both muscle tissues. (Figure 2C,D). Fragments with log2FC > 2 and *p* < 0.05 between the two groups of samples were identified as differentially expressed tRFs or tiRNAs (Figure 2E). tsRNAs in LD and PM exhibit distinct seed sequence characteristics (Figure 2F).

### 3.3. Enrichment Analysis of tRFs Differences in Testes Between LD and PM

**Figure 3:**
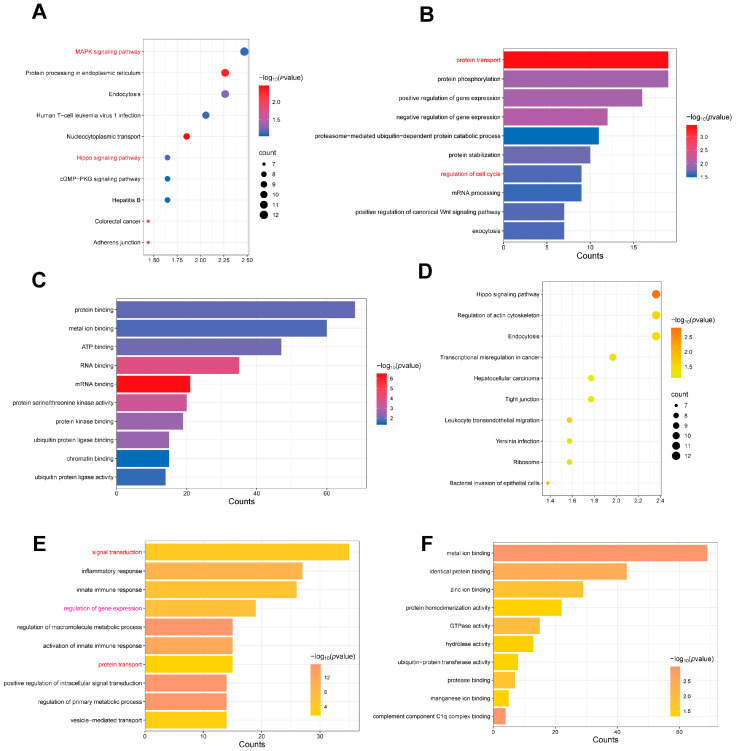
Enrichment analysis of the top 10 tsRNAs in the LD and PM of QingYu pigs. (**A**) The top 10 signaling pathways enriched with the target genes of the top 10 tsRNAs in PM. We have highlighted the key terms in red for emphasis. (**B**) The top 10 BP enriched with the target genes of the top 10 tsRNAs in PM. We have highlighted the key terms in red for emphasis. (**C**) The top 10 MF enriched with the target genes of the top 10 tsRNAs in PM. (**D**) The top 10 signaling pathways enriched with the target genes of the top 10 tsRNAs in LD. (**E**) The top 10 BP enriched with the target genes of the top 10 tsRNAs in LD. We have highlighted the key terms in red for emphasis. (**F**) The top 10 MF enriched with the target genes of the top 10 tsRNAs in LD.

To further investigate the potential functions of these differentially expressed tsRNAs, we performed enrichment analysis of the target genes of the top 10 most highly expressed tsRNAs in each group. The results showed that the target genes of the top 10 highly expressed tsRNAs in PM were mainly enriched in the MAPK signaling pathway, endocytosis, protein processing in the endoplasmic reticulum, cGMP-PKG signaling pathway, and Hippo signaling pathway (Figure 3A). BP enrichment analysis indicated that these target genes were primarily involved in post-translational modification, protein transport, cell cycle, and gene expression regulation (Figure 3B). Meanwhile, MF analysis revealed that these tsRNA target genes were mainly associated with protein binding and energy-related functions (Figure 3C), indicating a potential role in cellular signaling and metabolism. These findings suggest that the top 10 tsRNAs in PM may regulate the cell cycle by affecting protein homeostasis and energy metabolism, ultimately leading to abnormal cell proliferation.

Subsequently, we analyzed the target genes of the top 10 most highly expressed tsRNAs in LD. KEGG enrichment analysis showed that these target genes were enriched in the Hippo signaling pathway, regulation of actin cytoskeleton, and tight junction signaling pathways (Figure 3D). BP enrichment analysis highlighted the primary enrichment of these target genes in signaling transduction, gene expression regulation, and protein transport (Figure 3E). While MF analysis revealed enrichment in metal ion binding, identical protein binding, and zinc ion binding functions (Figure 3F).

### 3.4. tRF-Lys-TTT-012 Was Highly Expressed in PM

**Figure 4:**
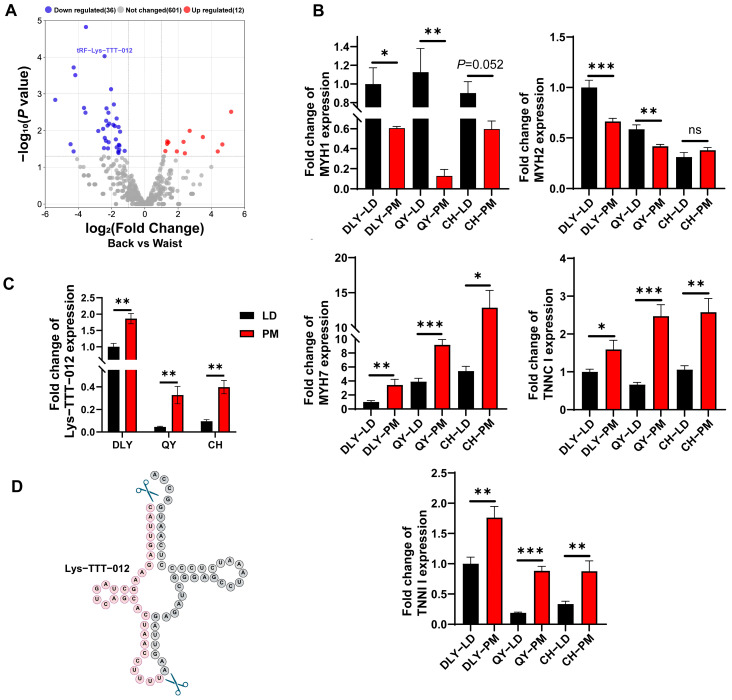
tRF-Lys-TTT-012 highly expressed in PM. (**A**) Volcano plot of differentially expressed tsRNAs in the LD and PMs. Lys-TTT-012 is highlighted in the plot. tRFs and tiRNAs are represented by red dots (indicating upregulation) or blue dots (indicating downregulation) above the dashed line, while gray dots represent tsRNAs with no significant differential expression. (**B**) The relative quantification of fast- and slow-twitch muscle fiber markers in the LD and PM of Duroc x Landrace x Yorkshire (DLY) pigs, Qingyu (QY) pigs, and Chenghua (CH) pigs was performed using qRT-PCR. (**C**) The quantification of tRF-Lys-TTT-012 was conducted in two muscle tissues from three pig species. (**D**) The shear site map of tRF-Lys-TTT-012 derived from tRNA-Lys-TTT. All data were presented as mean ± SEM. The symbol * denotes a significance level of * *p* < 0.05, ** *p* < 0.01, *** *p* < 0.001, ns indicates no significance.

Based on the Log2FC, *p*-value, Q-value in the sequencing results and the CPM values of the tsRNAs in the two muscle samples, we screened tRF-Lys-TTT-012 that were left behind after the sequencing results of the porcine LD and PM in order to carry out the subsequent functional validation (Figure 4A). We measured the level of Lys-TTT-012 in the muscle tissues of exotic pigs (DLY) and indigenous pigs (QY/CH) (Figure 4B,C). Based on the Lys-TTT-012 base sequence, we mapped its tRNA shear site, and Lys-TTT-012 originated from the 5′ end region of tRNA-Lys-TTT (Figure 4D), the so-called tRF-5c (Figure 2D).

### 3.5. Enrichment Analysis of tRF-Lys-TTT-012

**Figure 5:**
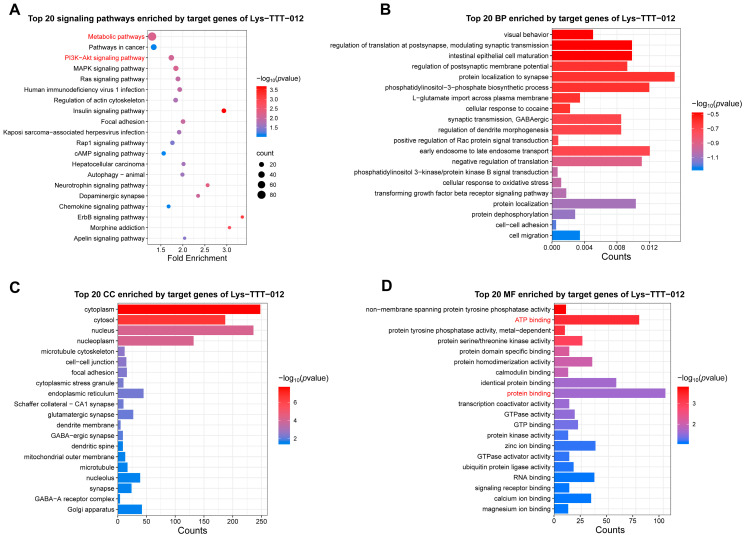
Enrichment analysis of target genes of tRF-Lys-TTT-012. (**A**) The top 20 signaling pathways enriched with the target genes of tRF-Lys-TTT-012. (**B**) The top 20 BP enriched with the target genes of tRF-Lys-TTT-012. (**C**) The top 20 CC enriched with the target genes of tRF-Lys-TTT-012. (**D**) The top 20 MF enriched with the target genes of tRF-Lys-TTT-012. We have highlighted the key terms in red for emphasis.

Based on the differences observed in tRF-Lys-TTT-012 between LD and PM, we performed functional enrichment analysis of its target genes to explore the potential impacts of these differences. KEGG analysis revealed significant enrichment in pathways related to metabolism, PI3K-AKT, and MAPK signaling, all of which are closely associated with energy metabolism (Figure 5A). BP enrichment analysis indicated that these target genes are primarily involved in biological processes such as protein localization and translation regulation (Figure 5B). CC analysis showed that these target genes primarily function in the cytoplasm (Figure 5C). Moreover, MF enrichment analysis suggested a strong association between these target genes and ATP binding as well as protein binding (Figure 5D). Together, these findings suggest that tRF-Lys-TTT-012 may influence protein function and energy metabolism, thereby affecting cell proliferation and muscle fiber differentiation.

### 3.6. Overexpression tRF-Lys-TTT-012 Inhibits Proliferation and Induces G1/S Phase Block in C2C12 Cells

**Figure 6:**
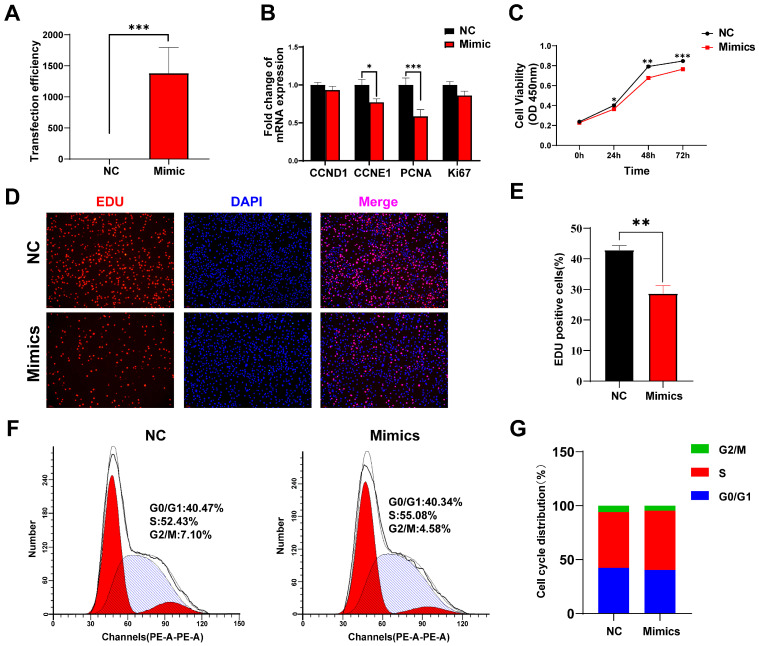
Overexpression tRF-Lys-TTT-012 inhibits proliferation of C2C12 myoblasts in vitro. (**A**) The transfection efficacy of tRF-Lys-TTT-012 mimics was validated by qRT-PCR. (**B**) The relative mRNA expression of cell proliferation-related genes after overexpression of tRF-Lys-TTT-012 in C2C12 cells. (**C**) CCK-8 assay was used to detect the proliferative activity of C2C12 cells transfected with tRF-Lys-TTT-012 mimics. (**D**) EDU assay was conducted to assess the effect of transfected tRF-Lys-TTT-012 mimics on the proliferation of C2C12 cells. Scale: 1 bar represents 100 μm. (**E**) Histogram showing quantitative results from EDU assay. ImageJ software counted the proportion of EDU-positive cells. (**F**,**G**) Flow cytometry results showed the effect of tRF-Lys-TTT-012 overexpression on the cell cycle of C2C12 myoblasts. All data were presented as Mean ± SEM. The symbol * denotes a significance level of *p* < 0.05, ** *p* < 0.01, *** *p* < 0.001. The relative mRNA levels are normalized to β-actin. NC, negative control; Mimics, tRF-Lys-TTT-012 mimics.

To investigate the function of tRF-Lys-TTT-012 in skeletal muscle, we designed RNA mimics of tRF-Lys-TTT-012 and transfected mimics into C2C12 myoblasts to increase tRF-Lys-TTT-012 expression in order to probe the biological function of tRF-Lys-TTT-012 in C2C12 myoblasts (Figure 6A). Building on our preliminary analysis of its potential functions, we assessed the impact of Lys-TTT-012 on the proliferation of C2C12 cells. The overexpression of tRF-Lys-TTT-012 significantly reduced the cell cycle protein E1 (CCNE1, *p* < 0.05) and resulted in a highly significant reduction in the mRNA levels of proliferating cell nuclear antigen (PCNA, *p* < 0.001). Moreover, for other proliferation-related genes such as CCND1 and Ki67, the mRNA levels also exhibited a decreasing trend (Figure 6B). CCK-8 and EDU assays further demonstrated that the overexpression of tRF-Lys-TTT-012 significantly inhibited the proliferative activity of C2C12 cells and reduced the number of proliferating cells (Figure 6C–E). Additionally, we investigated the cell cycle arrest of C2C12 cells using flow cytometry and observed that the overexpression of tRF-Lys-TTT-012 induced G1/S phase arrest in C2C12 myoblasts, leading to a decrease in cell proliferation viability. (Figure 6F,G). Collectively, our data results suggested that the overexpression of tRF-Lys-TTT-012 hindered C2C12 myoblasts proliferation, mainly attributed to the blockage of G1/S transition.

### 3.7. Overexpression of tRF-Lys-TTT-012 Promoted C2C12 Cells Differentiation

**Figure 7:**
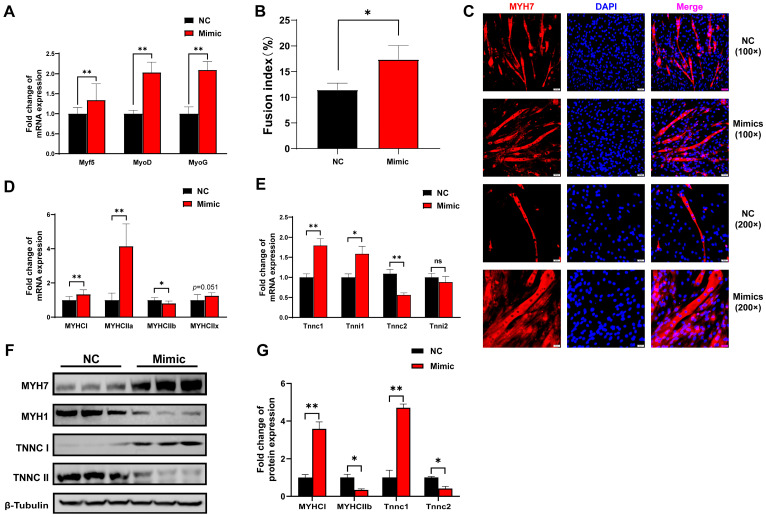
Overexpression of tRF-Lys-TTT-012 promotes the differentiation of C2C12 myoblasts and the transition to slow muscle fibers in vitro. (**A**) The effect of tRF-Lys-TTT-012 overexpression on the relative mRNA expression of myofiber type-related marker genes during C2C12 cell differentiation. (**B**) Histogram showing quantitative results of multinucleated myotubes in immunofluorescence assay. (**C**) Immunofluorescence staining of MYHC with anti-MYH7 antibody in C2C12 cells 6 days after differentiation. Scale: 1 bar represents 100 μm or 200 μm. The immunofluorescence images for Myh7 quantities were analyzed using ImageJ software. (**D**) The effect of tRF-Lys-TTT-012 overexpression on the relative mRNA expression of myofiber type marker genes during C2C12 cell differentiation. (**E**) The effect of tRF-Lys-TTT-012 overexpression on the relative mRNA expression of troponin marker genes during C2C12 cell differentiation. (**F**) The effect of tRF-Lys-TTT-012 overexpression on the relative protein expression of troponin marker genes during C2C12 cell differentiation. (**G**) Histogram showing quantitative results of the relative protein expression of troponin marker genes. All data were presented as Mean ± SEM. The symbol * denotes a significance level of *p* < 0.05, ** *p* < 0.01, ns indicates no significance. The relative mRNA levels are normalized to β-actin. NC, Negative control; Mimics, tRF-Lys-TTT-012 mimics.

Considering that tRF-Lys-TTT-012 was screened for functional validation based on its differential expression in different muscle types, we explored the effects of tRF-Lys-TTT-012 mimics on C2C12 myoblast differentiation and myofiber type transformation. After differentiating C2C12 myoblasts for 6 days with overexpression of tRF-Lys-TTT-012, we observed a notable increase in the mRNA levels of the myogenic differentiation marker genes Myf5, MyoD, and MyoG (Figure 7A). Immunofluorescence analysis of Myh7 demonstrated that tRF-Lys-TTT-012 mimics enhanced myotube formation during C2C12 cell differentiation, while increasing the incidence of cell fusion and multinucleated myotube formation events (Figure 7B,C). Given the potential association of tRF-Lys-TTT-012 with myofiber type conversion, we performed qRT-PCR to detect the mRNA expression of marker genes representing different myofiber types. The results demonstrated that the overexpression of tRF-Lys-TTT-012 upregulated the expression of slow muscle marker genes MYHCI and MYHCIIa at the mRNA level, while downregulating the expression of the fast muscle marker gene MYHCIIa. However, it had minimal effect on the intermediate-type myofibril marker gene MYHCIIx (Figure 7D). Considering that calcium release plays a crucial role in muscle contraction, similarly, we observed that overexpressing tRF-Lys-TTT-012 led to an increase in mRNA levels of troponin associated with slow muscle fibers (Figure 7E). Western Blot analysis showed that the protein levels of slow muscle fiber marker genes were consistent with the changes in mRNA levels and were also regulated by tRF-Lys-TTT-012 (Figure 7F,G). All these results collectively indicate that tRF-Lys-TTT-012 mimics promotes the differentiation of myofibroblasts while also promoting the transformation of myofibrillar dimensions into slow muscle fibers.

### 3.8. Overexpression of tRF-Lys-TTT-012 Affected Mitochondrial Function

**Figure 8:**
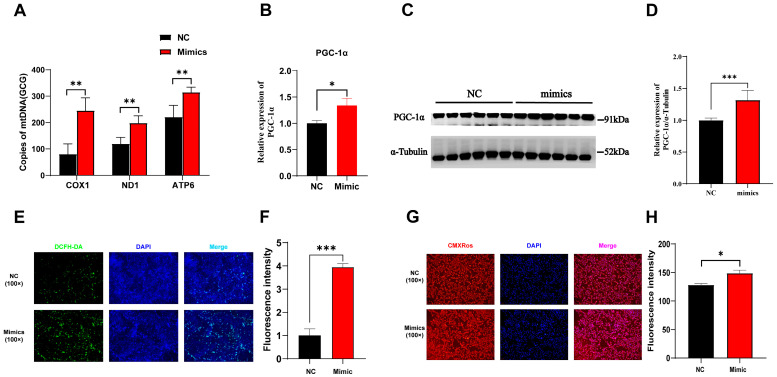
Overexpression of tRF-Lys-TTT-012 increases mitochondrial content and promotes mitochondrial function in vitro. (**A**) The effect of mitochondrial copy number after overexpression of Lys-TTT-012. (**B**) The relative mRNA expression of PGC-1α after tRF-Lys-TTT-012 overexpression in C2C12 cells. (**C**,**D**) The change in protein levels of PGC-1α after overexpression of tRF-Lys-TTT-012. (**E**) Immunofluorescence analysis depicting the effect of tRF-Lys-TTT-012 overexpression on fluorescence intensity related to mitochondrial reactive oxygen species in PK15 cells. (**F**) Quantification of fluorescence intensity corresponding to mitochondrial reactive oxygen species staining in PK15 cells with tRF-Lys-TTT-012 overexpression using the ImageJ software. (**G**) Mitochondrial fluorescence staining showing the impact of tRF-Lys-TTT-012 overexpression on mitochondrial content within PK15 cells. (**H**) Quantification of mitochondrial content using the ImageJ software based on fluorescence intensity in PK15 cells with tRF-Lys-TTT-012 overexpression. All data were presented as Mean ± SEM. The symbol * denotes a significance level of *p* < 0.05, ** *p* < 0.01, *** *p* < 0.001. The relative mRNA levels are normalized to β-actin. NC, negative control; Mimics, tRF-Lys-TTT-012 mimcis.

Previous studies have highlighted the critical role of PGC1α in mitochondrial biogenesis and muscle fiber type transformation [22]. The mitochondrial copy number serves as a direct indicator of mitochondrial abundance and functional capacity. Our results demonstrated that the overexpression of tRF-Lys-TTT-012 led to an increase in the expression of three genes (COX1, ND1, ATP6) related to mitochondrial copy number (Figure 8A). PGC1α as a pivotal transcription factor for mitochondrial biogenesis [23]. We demonstrated that the overexpression of tRF-Lys-TTT-012 had a positive impact on enhancing both its mRNA and protein levels (Figure 8B–D). Additionally, immunofluorescence analysis revealed that the overexpression of tRF-Lys-TTT-012 in PK15 cells resulted in an elevation of the fluorescence intensity in the staining specific to mitochondrial reactive oxygen species (Figure 8E,F). Similarly, mitochondrial fluorescence staining demonstrated that the overexpression of tRF-Lys-TTT-012 also led to an increase in mitochondrial content within PK15 cells (Figure 8G,H). In summary, these results demonstrate that the overexpression of tRF-Lys-TTT-012 mediates the transition of muscle fiber type to slow muscle by increasing the transcript and protein levels of PGC1α and thereby promoting mitochondrial functioning.

## 4. Discussion

In this experiment, we acquired the tissue-specific expression profiles of tsRNAs in two distinct muscle tissues through high-throughput sequencing of porcine LD and PM. The primary distinction between the two muscle tissues, namely the LD and PM, resides in their distinct composition of muscle fiber types. The LD is predominantly composed of fast muscle fibers, primarily reliant on glycolysis to fulfill energy requirements. Conversely, the PM mainly consists of slow muscle fibers that primarily depend on oxidative metabolism for energy supply. Research has demonstrated that the metabolic distinctions between fast and slow muscles arise from the greater abundance of mitochondria and a denser mitochondrial ultrastructure in slow muscle fibers [24,25]. This structural arrangement enables predominant energy provisioning through oxidative metabolism, thereby sustaining elevated endurance levels in slow muscles. Additionally, different muscle fiber types also have different effects on meat quality. The prevailing scientific consensus asserts that muscles possessing a larger ratio of slow or oxidized fibers (MyHC I and MyHC IIa) play a crucial role in enhancing meat tenderness and flavor, in contrast to muscles primarily comprising fast or glycolytic fibers [26,27,28,29].

Interconversion between distinct muscle fiber types is a feasible phenomenon. The increasing body of evidence underscores the participation of non-coding RNAs in the intricate biological process of muscle fiber type switching [30,31,32]. For instance, miR-22-3p has the capacity to hinder the shift in skeletal muscle fibers from fast-twitch to slow-twitch induced by resveratrol [31]. Knockout of MiR-208b resulted in the suppression of slow muscle fiber development in both C2C12 cells and mice. Conversely, the inhibition of Mettl8 (Methyltransferase like 8), the target gene of MiR-208b, produced effects opposite to those of miR-208b [33]. These studies are consistent with the emerging understanding that non-coding RNAs play pivotal roles in regulating various cellular processes, including those related to muscle physiology. tsRNAs, as emerging non-coding RNAs, have captured considerable attention in recent years. However, the bulk of existing research in this realm has predominantly focused on unraveling their roles in cancer and various disease contexts [34,35]. In contrast, investigations into other areas, such as myofiber type conversion, are still in their early stages.

Due to the scarcity of research studies concerning tsRNAs in the realm of skeletal muscle growth, development, and muscle fiber type conversion, we proceeded to analyze the sequencing results. Interestingly, we performed RNA quantification of tRF-Lys-TTT-012 and muscle fiber markers in exotic lean-type pigs (DLY) and indigenous obese-type pigs (QY/CH). We found the most significant differences in the content of oxidative and glycolytic muscle fibers between the LD and PMs of QY pigs, with the largest consistency in the differential expression of tRF-Lys-TTT-012, reaching up to fivefold. Notably, we identified tRF-Lys-TTT-012, exhibiting high expression in the PM, for subsequent functional investigation. Taking into consideration the significant conservation of tRF-Lys-TTT-012 across different species and the suitability of the C2C12 cell line for researching muscle development and myofiber type conversion, chose C2C12 myoblasts to intensively investigate the role of tRF-Lys-TTT-012 in the process of myofiber type conversion in vitro [36]. Upon overexpressing tRF-Lys-TTT-012, we observed a noticeable reduction in the proliferative capacity of C2C12 myoblasts. This effect was primarily attributed to G1/S phase arrest, as evidenced by flow cytometry analysis. Moreover, when the cell density of C2C12 myoblasts reaches 80%, a sequence of cell fusion events is triggered, subsequently leading to their progressive differentiation into myotubes under the induction and cultivation of low-nutrient conditions (2% horse serum). Further analyses revealed that tRF-Lys-TTT-012 promoted the differentiation of C2C12 myoblasts to multinucleated myotubes. Additionally, it was observed that the expression of genes associated with slow myofibrillar development was enhanced during this process. These findings suggest that overexpression of tRF-Lys-TTT-012 not only promotes the differentiation of C2C12 myoblasts but also facilitates the transition from myofibrillar to slow myofibrillar generation.

There is evidence indicating variations in the mitochondrial content among different muscle fiber types [37]. Slow muscle fibers, for instance, typically exhibit a higher mitochondrial abundance compared to other fiber types [25]. Simultaneously, the process of myofiber type conversion involves the regulation of mitochondrial biogenesis to fulfill the increased energy requirements. PGC1α, serving as a pivotal transcription factor in mitochondrial biogenesis, plays a significant role in governing both mitochondrial quantity and their metabolic functionalities [22]. In this experiment, we observed that tRF-Lys-TTT-012 overexpression led to an elevation in PGC1α expression, evident not only at the mRNA level but also at the protein level. Simultaneously, through analysis of mitochondrial copy number-associated genes and immunofluorescence staining of mitochondria, a notable increase in mitochondrial content was identified. The available evidence indicates that a slight elevation in reactive oxygen species (ROS) levels initiates the activation of the PGC-1alpha (peroxisome proliferator-activated receptor gamma coactivator 1 alpha) signaling pathway. In turn, PGC1 activation improves both the quantity and functionality of mitochondria. This concept effectively elucidates why the overexpression of tRF-Lys-TTT-012 resulted in not only an increase in mitochondrial content but also a subtle enhancement in the immunofluorescence intensity of cellular reactive oxygen species.

Unfortunately, in our study, our understanding of the mechanisms regulating mitochondrial quantity, quality, and the key factor PGC1α has remained at the current stage. We were unable to determine the further association between tsRNAs and mitochondrial biogenesis. In the mitochondrial quality control (MQC) program, PGC1α is intricately and orderly involved in interactions with mitochondrial autophagy and mitochondrial fusion-fission processes [38,39]. In-depth exploration of the relationship between these mitochondrial regulatory processes and tsRNAs will help expand our understanding of the mechanisms underlying muscle fiber type conversion.

Overall, our findings suggest that tRF-Lys-TTT-012 plays a role in regulating muscle fiber type transition in C2C12 cells. However, it is important to acknowledge that our in vitro findings in C2C12 cells may not fully replicate the complex in vivo environment. In vivo, factors such as tissue-specific interactions, the extracellular matrix, and systemic signaling pathways could influence the effects of tRF-Lys-TTT-012, which may not be fully captured in the cell culture model used in this study. Therefore, further validation in animal models is essential to confirm the physiological relevance of our findings. Our bioinformatic analysis predicts several potential targets of tRF-Lys-TTT-012; however, it is important to note that tsRNA target prediction is still an evolving field, and current methods often face challenges in accurately predicting biological interactions. These predictions are based on computational algorithms, which, while helpful, still require experimental validation to confirm the exact mechanisms. Therefore, further experimental validation using techniques such as RNA immunoprecipitation (RIP) and reporter assays is necessary to confirm these predicted interactions.

## 5. Conclusions

In summary, tRF-Lys-TTT-012 is highly expressed in the predominantly slow muscle of the PM. Additionally, tRF-Lys-TTT-012 overexpression reduces C2C12 proliferation and encourages myogenic differentiation. It also has a significant impact on mitochondrial content and function by influencing PGC1, a crucial transcription factor for mitochondrial biogenesis, which causes myofibers to change into slow muscle fibers. These findings underscore the critical role of tRF-Lys-TTT-012 in modulating mitochondrial dynamics and muscle fiber type transitions, shedding light on potential avenues for further research in enhancing muscle performance and metabolic adaptation.

## Figures and Tables

**Table 1:** The sequences of exogenous mimics.

Names	Sequence 5′-3′
Mimics NC	UUGUACUACACAAAAGUACUG
tRF-Lys-TTT-012 mimics	CAUUGAGAAGCUAGUCAGCACUAACCUUUU

**Table 2:** The primer sequences for qRT-PCR.

Names	Primer	Sequence 5′-3′	Melting Temperature (°C)
mmu-MyoD	Forward	CCACTCCGGGACATAGACTTG	57
	Reverse	AAAAGCGCAGGTCTGGTGAG	
mmu-MyoG	Forward	GAGACATCCCCCTATTTCTACCA	58
	Reverse	GCTCAGTCCGCTCATAGCC	
mmu-PGC1α	Forward	TATGGAGTGACATAGAGTGTGCT	60
	Reverse	CCACTTCAATCCACCCAGAAAG	
mmu-Myf5	Forward	CGGATCACGTCTACAGAGCC	57
	Reverse	GCAGGAGTGATCATCGGGAG	
mmu-MyHC Ι	Forward	GGCCCCTTCCAGCTTGA	60.5
	Reverse	TGGCTGCGCCTTGGTTT	
mmu-MyHC ΙΙa	Forward	TTAAAAAGCTCCAAGAACTGTTTCA	60
	Reverse	CCATTTCCTGGTCGGAACTC	
mmu-MyHC ΙΙb	Forward	CACTTTAAGTAGTTGTCTGCCTTGAG	60
	Reverse	GGCAGCAGGGCACTAGATGT	
mmu-MyHC ΙΙx	Forward	AGCTTCAAGTTCTGCCCCACT	60
	Reverse	GGCTGCGGGTTATTGATGG	
mmu-Tnnc1	Forward	ATGGTTCGGTGCATGAAGGA	61
	Reverse	ATCCTCTGTGATGGTCTCGC	
mmu-Tnni1	Forward	CCCCACAGTCTGCAGTCCA	60
	Reverse	CTCTCAACTTCCGGCATGGT	
mmu-Tnnc2	Forward	CCGCCTTCGACATGTTTGAC	60
	Reverse	TCGATGGTTCCACTGCCATC	
mmu-Tnni2	Forward	TGTTCGACCTGAGAGGCAAG	58
	Reverse	GCACACTTTGTGCTTGGAGC	
mmu-CCND1	Forward	GCGTACCCTGACACCAATCTC	60
	Reverse	CTCCTCTTCGCACTTCTGCTC	
mmu-CCNE1	Forward	GTGGCTCCGACCTTTCAGTC	60
	Reverse	CACAGTCTTGTCAATCTTGGCA	
ssc-ATP6	Forward	TATTTGCCTCTTTATTGCCC	58
	Reverse	GGATCGAGATTGTGCGGTTAT	
ssc-ND1	Forward	GCCACATCCTCAATCTCCAT	59
	Reverse	GATTAGAGGGTAGGGTATTGGTAG	
ssc-COX1	Forward	ACTACTGACAGACCGCAACC	60
	Reverse	TCCAATGGACATTATGGCTC	
ssc-GCG	Forward	GAATCAACACCATCGGTCAAAT	60
	Reverse	CTCCACCCATAGAATGCCCAGT	
ssc-ATP8	Forward	CATTCCCACTGGCACCTTCA	60
	Reverse	TGAGGCAAATAGATTTTCGTTCA	
ssc-β-actin	Forward	TCAGCAAGCAGGAGTACGAC	60
	Reverse	TCACAGCTTCTCAGCAGACAG	
ssc-GAPDH	Forward	GTCGGAGTGAACGGATTTGGC	60
	Reverse	CACCCCATTTGATGTTGGCG	
mmu-actin	Forward	GCTGTATTCCCCTCCATCGT	60
	Reverse	CTTCTCCATGTCGTCCCAGT	
ssc-Lys-TTT-012	Forward	CATTGAGAAGCTAGTCAGCACTAACCTTTT	60
	Reverse	Uni-miR qPCR Primer, included in kit (TaKaRa)	
U6	Forward	CTCGCTTCGGCAGCACA	60
	Reverse	AACGCTTCACGAATTTGCGT	

## Data Availability

The original contributions presented in this study are included in the article. Further inquiries can be directed to the corresponding authors.

## Abbreviations

The following abbreviations are used in this manuscript:

tRFs	tRNA-derived fragments
MYHC	Myosin heavy chains
LD	Latissimus dorsi muscle
PM	Psoas major muscle

## References

[1] Dumont N.A., Bentzinger C.F., Sincennes M.-C., Rudnicki M.A. (2015). Satellite Cells and Skeletal Muscle Regeneration. Compr. Physiol..

[2] Schiaffino S., Reggiani C. (2011). Fiber Types in Mammalian Skeletal Muscles. Physiol. Rev..

[3] Schiaffino S. (2018). Muscle Fiber Type Diversity Revealed by Anti-Myosin Heavy Chain Antibodies. FEBS J..

[4] Qaisar R., Bhaskaran S., Van Remmen H. (2016). Muscle Fiber Type Diversification during Exercise and Regeneration. Free Radic. Biol. Med..

[5] Ying F., Zhang L., Bu G., Xiong Y., Zuo B. (2016). Muscle Fiber-Type Conversion in the Transgenic Pigs with Overexpression of PGC1α Gene in Muscle. Biochem. Biophys. Res. Commun..

[6] Wu L., Ran L., Lang H., Zhou M., Yu L., Yi L., Zhu J., Liu L., Mi M. (2019). Myricetin Improves Endurance Capacity by Inducing Muscle Fiber Type Conversion via miR-499. Nutr. Metab..

[7] Zhang D., Wang X., Li Y., Zhao L., Lu M., Yao X., Xia H., Wang Y.-C., Liu M.-F., Jiang J. (2014). Thyroid Hormone Regulates Muscle Fiber Type Conversion via miR-133a1. J. Cell Biol..

[8] Gan M., Shen L., Liu L., Guo Z., Wang S., Chen L., Zheng T., Fan Y., Tan Y., Jiang D. (2020). miR-222 Is Involved in the Regulation of Genistein on Skeletal Muscle Fiber Type. J. Nutr. Biochem..

[9] Yu X., Xie Y., Zhang S., Song X., Xiao B., Yan Z. (2021). tRNA-Derived Fragments: Mechanisms Underlying Their Regulation of Gene Expression and Potential Applications as Therapeutic Targets in Cancers and Virus Infections. Theranostics.

[10] Weng Q., Wang Y., Xie Y., Yu X., Zhang S., Ge J., Li Z., Ye G., Guo J. (2022). Extracellular Vesicles-Associated tRNA-Derived Fragments (tRFs): Biogenesis, Biological Functions, and Their Role as Potential Biomarkers in Human Diseases. J. Mol. Med..

[11] Kumar P., Kuscu C., Dutta A. (2016). Biogenesis and Function of Transfer RNA-Related Fragments (tRFs). Trends Biochem. Sci..

[12] Shen L., Liao T., Chen Q., Lei Y., Wang L., Gu H., Qiu Y., Zheng T., Yang Y., Wei C. (2023). tRNA-Derived Small RNA, 5’tiRNA-Gly-CCC, Promotes Skeletal Muscle Regeneration through the Inflammatory Response. J. Cachexia Sarcopenia Muscle.

[13] Zhu X.-L., Li T., Cao Y., Yao Q.-P., Liu X., Li Y., Guan Y.-Y., Deng J.-J., Jiang R., Jiang J. (2021). tRNA-Derived Fragments tRFGlnCTG Induced by Arterial Injury Promote Vascular Smooth Muscle Cell Proliferation. Mol. Ther. Nucleic Acids.

[14] Sobala A., Hutvagner G. (2013). Small RNAs Derived from the 5’ End of tRNA Can Inhibit Protein Translation in Human Cells. RNA Biol..

[15] Gan M., Ma J., Chen J., Chen L., Zhang S., Zhao Y., Niu L., Li X., Zhu L., Shen L. (2022). miR-222 Is Involved in the Amelioration Effect of Genistein on Dexamethasone-Induced Skeletal Muscle Atrophy. Nutrients.

[16] Cheng X., Du J., Shen L., Tan Z., Jiang D., Jiang A., Li Q., Tang G., Jiang Y., Wang J. (2018). MiR-204-5p Regulates C2C12 Myoblast Differentiation by Targeting MEF2C and ERRγ. Biomed. Pharmacother..

[17] Shen L., Chen L., Zhang S., Du J., Bai L., Zhang Y., Jiang Y., Li X., Wang J., Zhu L. (2016). MicroRNA-27b Regulates Mitochondria Biogenesis in Myocytes. PLoS ONE.

[18] Wang Q., Lee I., Ren J., Ajay S.S., Lee Y.S., Bao X. (2013). Identification and Functional Characterization of tRNA-Derived RNA Fragments (tRFs) in Respiratory Syncytial Virus Infection. Mol. Ther..

[19] Sharma U., Conine C.C., Shea J.M., Boskovic A., Derr A.G., Bing X.Y., Belleannee C., Kucukural A., Serra R.W., Sun F. (2016). Biogenesis and Function of tRNA Fragments during Sperm Maturation and Fertilization in Mammals. Science.

[20] Gan M., Ma J., Chen L., Zhang S., Niu L., Zhao Y., Li X., Pan H., Zhu L., Shen L. (2022). Identification of tRNA-Derived Small RNAs and Their Potential Roles in Porcine Skeletal Muscle with Intrauterine Growth Restriction. Front. Physiol..

[21] Gu H., Gan M., Wang L., Yang Y., Wang J., Chen L., Zhang S., Zhao Y., Niu L., Jiang D. (2022). Differential Expression Analysis of tRNA-Derived Small RNAs from Subcutaneous Adipose Tissue of Obese and Lean Pigs. Animals.

[22] Selsby J.T., Morine K.J., Pendrak K., Barton E.R., Sweeney H.L. (2012). Rescue of Dystrophic Skeletal Muscle by PGC-1α Involves a Fast to Slow Fiber Type Shift in the Mdx Mouse. PLoS ONE.

[23] Kong S., Cai B., Nie Q. (2022). PGC-1α Affects Skeletal Muscle and Adipose Tissue Development by Regulating Mitochondrial Biogenesis. Mol. Genet. Genom..

[24] Murgia M., Nagaraj N., Deshmukh A.S., Zeiler M., Cancellara P., Moretti I., Reggiani C., Schiaffino S., Mann M. (2015). Single Muscle Fiber Proteomics Reveals Unexpected Mitochondrial Specialization. EMBO Rep..

[25] Gouspillou G., Sgarioto N., Norris B., Barbat-Artigas S., Aubertin-Leheudre M., Morais J.A., Burelle Y., Taivassalo T., Hepple R.T. (2014). The Relationship between Muscle Fiber Type-Specific PGC-1α Content and Mitochondrial Content Varies between Rodent Models and Humans. PLoS ONE.

[26] Choe J.H., Choi Y.M., Lee S.H., Shin H.G., Ryu Y.C., Hong K.C., Kim B.C. (2008). The Relation between Glycogen, Lactate Content and Muscle Fiber Type Composition, and Their Influence on Postmortem Glycolytic Rate and Pork Quality. Meat Sci..

[27] Lee S.H., Kim J.-M., Ryu Y.C., Ko K.S. (2016). Effects of Morphological Characteristics of Muscle Fibers on Porcine Growth Performance and Pork Quality. Korean J. Food Sci. Anim. Resour..

[28] Matarneh S.K., Silva S.L., Gerrard D.E. (2021). New Insights in Muscle Biology That Alter Meat Quality. Annu. Rev. Anim. Biosci..

[29] Joo S.T., Kim G.D., Hwang Y.H., Ryu Y.C. (2013). Control of Fresh Meat Quality through Manipulation of Muscle Fiber Characteristics. Meat Sci..

[30] Jiang A., Dong C., Li B., Zhang Z., Chen Y., Ning C., Wu W., Liu H. (2019). MicroRNA-206 Regulates Cell Proliferation by Targeting G6PD in Skeletal Muscle. FASEB J..

[31] Wen W., Chen X., Huang Z., Chen D., Chen H., Luo Y., He J., Zheng P., Yu J., Yu B. (2020). Resveratrol Regulates Muscle Fiber Type Conversion via miR-22-3p and AMPK/SIRT1/PGC-1α Pathway. J. Nutr. Biochem..

[32] Cao H., Liu J., Du T., Liu Y., Zhang X., Guo Y., Wang J., Zhou X., Li X., Yang G. (2022). Circular RNA Screening Identifies circMYLK4 as a Regulator of Fast/Slow Myofibers in Porcine Skeletal Muscles. Mol. Genet. Genom..

[33] Li X., Bi H., Xie S., Cui W. (2022). MiR-208b Regulates the Conversion of Skeletal Muscle Fiber Types by Inhibiting Mettl8 Expression. Front. Genet..

[34] Cui H., Li H., Wu H., Du F., Xie X., Zeng S., Zhang Z., Dong K., Shang L., Jing C. (2022). A Novel 3’tRNA-Derived Fragment tRF-Val Promotes Proliferation and Inhibits Apoptosis by Targeting EEF1A1 in Gastric Cancer. Cell Death Dis..

[35] Han L., Lai H., Yang Y., Hu J., Li Z., Ma B., Xu W., Liu W., Wei W., Li D. (2021). A 5’-tRNA Halve, tiRNA-Gly Promotes Cell Proliferation and Migration via Binding to RBM17 and Inducing Alternative Splicing in Papillary Thyroid Cancer. J. Exp. Clin. Cancer Res..

[36] Zhang Y., Yang M., Zhou P., Yan H., Zhang Z., Zhang H., Qi R., Liu J. (2020). β-Hydroxy-β-Methylbutyrate-Induced Upregulation of miR-199a-3p Contributes to Slow-To-Fast Muscle Fiber Type Conversion in Mice and C2C12 Cells. J. Agric. Food Chem..

[37] Liu X., Trakooljul N., Hadlich F., Murani E., Wimmers K., Ponsuksili S. (2017). Mitochondrial-Nuclear Crosstalk, Haplotype and Copy Number Variation Distinct in Muscle Fiber Type, Mitochondrial Respiratory and Metabolic Enzyme Activities. Sci. Rep..

[38] Yeo D., Kang C., Gomez-Cabrera M.C., Vina J., Ji L.L. (2019). Intensified Mitophagy in Skeletal Muscle with Aging Is Downregulated by PGC-1alpha Overexpression in Vivo. Free Radic. Biol. Med..

[39] Tong M., Mukai R., Mareedu S., Zhai P., Oka S., Huang C.-Y., Hsu C.-P., Yousufzai F.A.K., Fritzky L., Mizushima W. (2023). Distinct Roles of DRP1 in Conventional and Alternative Mitophagy in Obesity Cardiomyopathy. Circ. Res..

